# Polysaccharide from *Lentinus edodes* Inhibits the Immunosuppressive Function of Myeloid-Derived Suppressor Cells

**DOI:** 10.1371/journal.pone.0051751

**Published:** 2012-12-18

**Authors:** Hao Wu, Ning Tao, Xiaoman Liu, Xiao Li, Jian Tang, Chungwah Ma, Xiaofei Xu, Haitao Shao, Baidong Hou, Hui Wang, Zhihai Qin

**Affiliations:** 1 Xinxiang Medical University, Xinxiang, China; 2 Protein and Peptide Pharmaceutical Laboratory, Institute of Biophysics, Chinese Academy of Sciences, Beijing, China; 3 Infinitus (China) Company Ltd., Guangzhou, China; 4 Key Laboratory of Infection and Immunity, Institute of Biophysics, Chinese Academy of Sciences, Beijing, China; Instituto Butantan, Brazil

## Abstract

Reversing the function of immune suppressor cells may improve the efficacy of cancer therapy. Here, we have isolated a novel polysaccharide MPSSS (577.2 Kd) from *Lentinus edodes* and examined its effects on differentiation and function of myeloid-derived suppressor cells (MDSCs). MPSSS is composed of glucose (75.0%), galactose (11.7%), mannose (7.8%), and xylose (0.4%). In vivo, it inhibits the growth of McgR32 tumor cells, which is correlated with a reduced percentage of MDSCs in peripheral blood. In vitro, it induces both morphological and biophysical changes in MDSCs. Importantly, MPSSS up-regulates MHC II and F4/80 expression on MDSCs, and reverses their inhibition effect on CD4^+^ T cells in a dose-dependent manner. The mechanism study shows that MPSSS may stimulate MDSCs through a MyD88 dependent NF-κB signaling pathway. Together, we demonstrated for the first time that MPSSS stimulates the differentiation of MDSCs and reverses its immunosuppressive functions, shedding new light on developing novel anti-cancer strategies by targeting MDSCs.

## Introduction

Myeloid-derived suppressor cells (MDSCs) are a group of Gr1^+^CD11b^+^ cells and were first identified in tumor bearing mice. Increasing evidence suggests that they are important immune regulatory cells. In human, the major function of MDSCs during tumor progression is to inhibit T cell activity and promote tumor growth [1]–[6]. Preventing the generation of MDSCs or inhibiting their function may promote anti-tumor immunity, and could be a new strategy for cancer treatment.

Polysaccharides from *L. edodes* exhibit strong anti-cancer activities [7], [8], which are host-mediated and associated with the activation of immune cells such as T cells, natural killer cells and macrophages [9]–[12]. However, whether polysaccharides from *L. edodes* are able to reduce immunological suppression in tumor, especially by MDSCs, is still unknown.

In this study, we isolated a novel polysaccharide component (MPSSS) from *L. edodes* and investigated its effect on MDSCs. We found that MPSSS induced MDSC differentiation and prevented their immunosuppressive function. This reveals a novel mechanism underlying the anti-tumor activity of polysaccharides and indicates that MPSSS is a potential candidate for anti-tumor therapy.

## Results

### Purification of MPSSS

**Figure 1:**
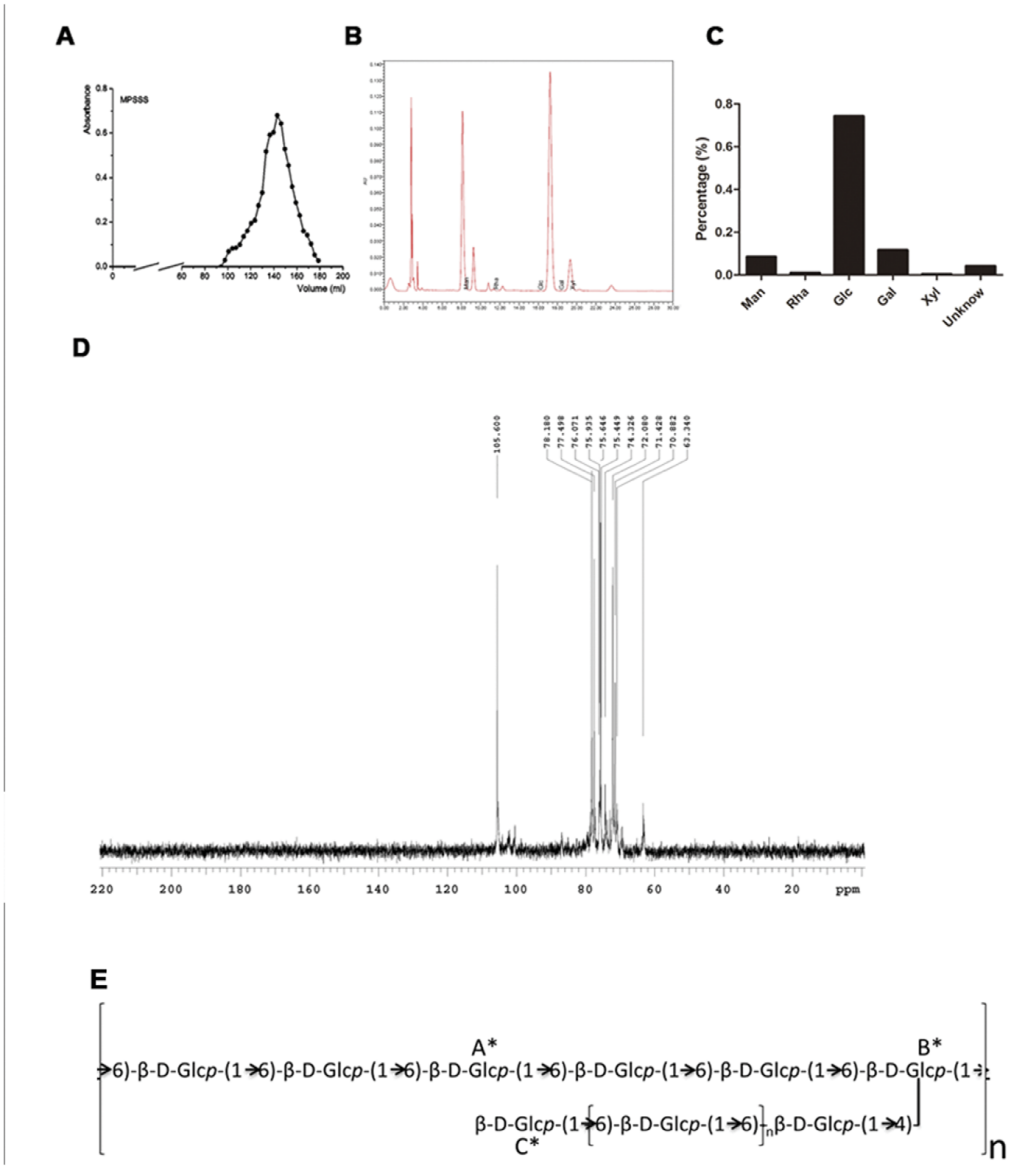
Composition and structure analysis of MPSSS. (A) MPSSS appeared as a single peak in Sepharose CL-6B chromatography, indicating its high degree of purity. (B) The monosaccharides derived from MPSSS were detected by HPLC. (C) Analytical results of the composition of MPSSS. (D) Results of ^13^C NMR detection of MPSSS. (E) Analytical results of the MPSSS polysaccharide basic structure unit.

*L. edodes* was extracted with hot water to obtain a total water soluble polysaccharide extract, and it was precipitated sequentially with 30%, 50% and 70% ethanol to obtain three pure fractions: MPPP, MPSSP2 and MPSSS. Given that MPSSS had the strongest stimulating effect on splenocytes, this fraction was used in the study. The yield of MPSSS was low, at only 1% of the total dry weight. The homogeneity and molecular weight of MPSSS were determined by size-exclusion chromatography and it gave a single symmetrical narrow peak on a Shodex SB column, with a molecular weight of 5.772×10^5^ Daltons (Da). Analysis of its monosaccharide composition showed that this polysaccharide fraction was mainly composed of glucose (75.0%), galactose (11.7%) and mannose (7.8%) (Fig. 1B, C). Its polymer fraction mainly consists of a β-1, 6-linked glucan branched at C-4 with side chains which are also β-1, 6-linked-glucans (Fig. 1 D, E; Table S1 in Supplemental material).

### MPSSS Inhibited Tumor Growth in vivo

**Figure 2:**
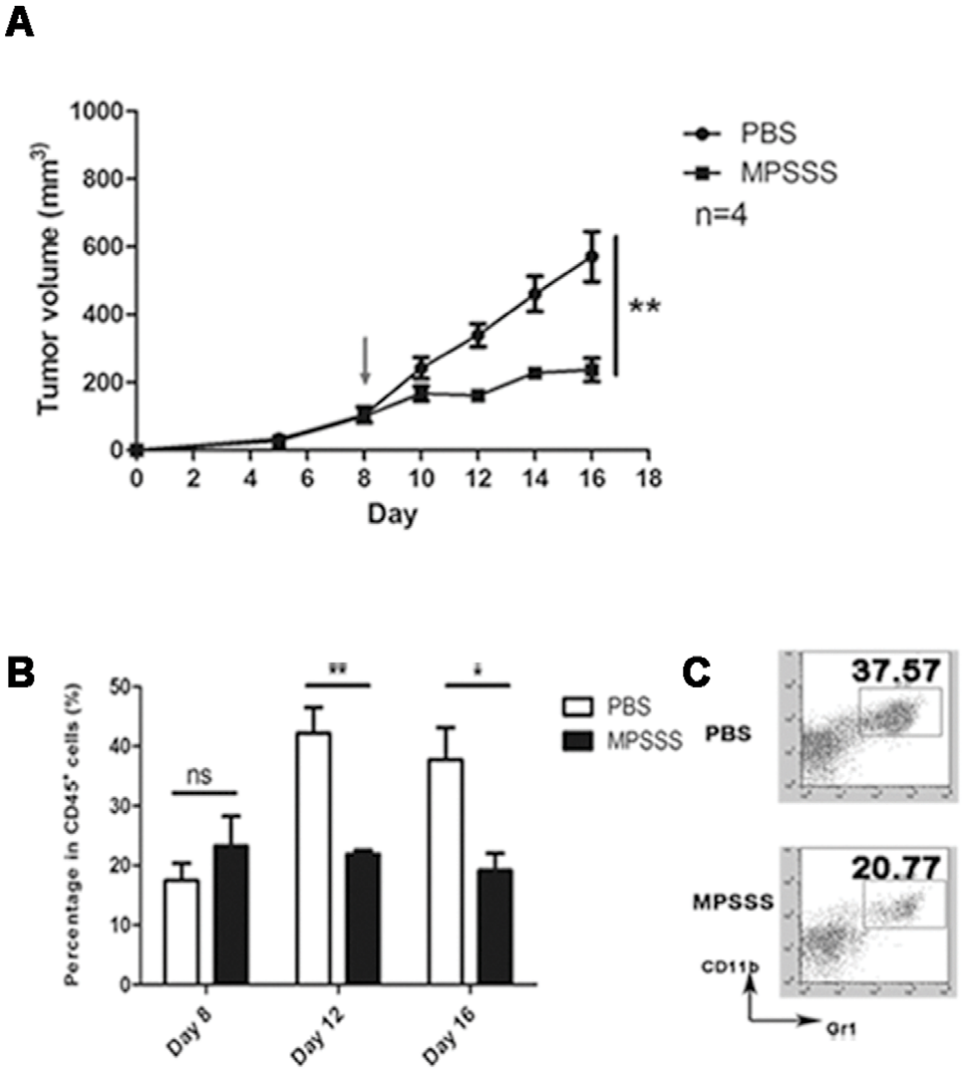
MPSSS inhibited tumor growth and reduced MDSC numbers. (A) C57BL/6 mice were injected subcutaneously with 1×10^6^ McgR32 cellsand 8 day later, with MPSSS or PBS intraperitoneally once every two days. Tumor volume was recorded as the mean ± SD and each group contained 3–4 mice and was analyzed by one-way ANOVA for repeated measures data. (B) At the indicated days after tumor cell injection, peripheral blood was taken for MDSC detection by flow cytometry, Quantitative data are expressed as mean ± SD and analyzed by Student's *t* tests. *P<0.05, **P<0.01. (C) A representative flow cytometry analysis was shown for the staining of CD11b and Gr1.

To test whether MPSSS has anti-tumor activity, we first examined its effect on the growth of McgR32 tumor cells in mice. As shown in Fig. 2A, tumors from the MPSSS-treated group grew slower and were significantly smaller (200 mm^3^) than those in the control group (580 mm^3^) at day 16. Respectively, the percentage of MDSCs in the peripheral blood was also significantly lower in the MPSSS group (19%) (Fig. 2B, C) compared to the control group (38%) at the same time. This result demonstrated that MPSSS inhibited tumor growth, which was accompanied by a decrease of MDSCs in mice.

### MPSSS Promoted Differentiation of MDSCs

**Figure 3:**
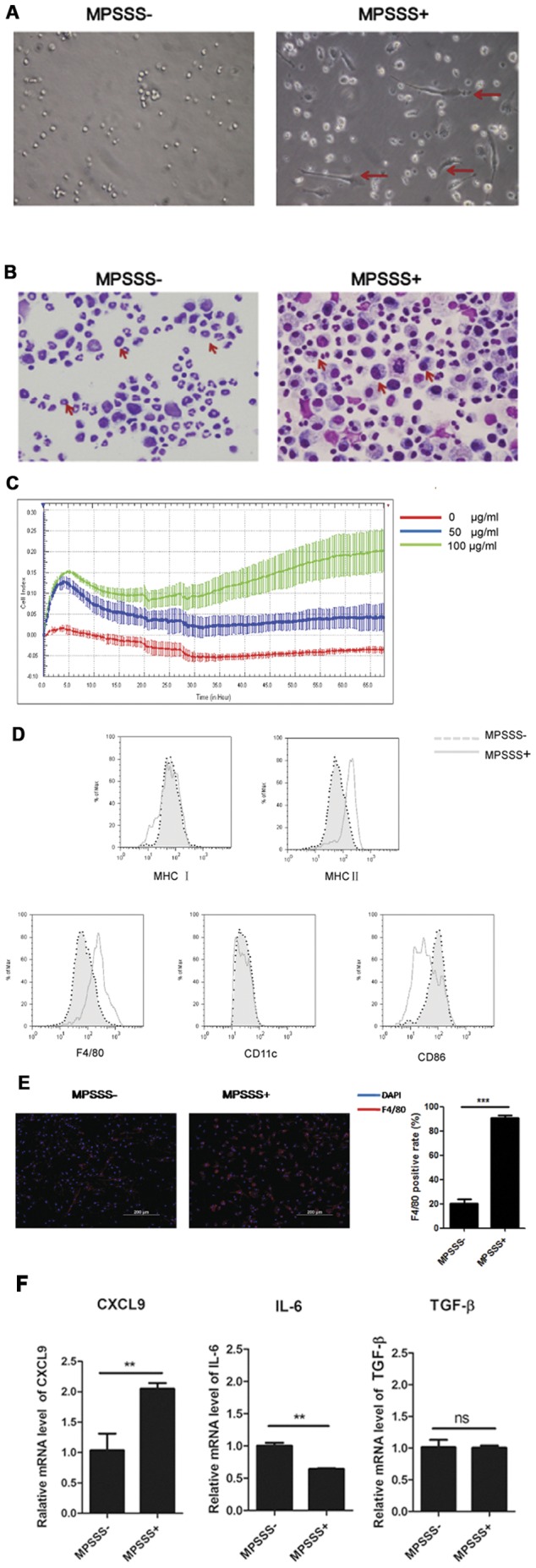
MPSSS promoted differentiation of MDSCs. (A) FACS-sorted splenic MDSCs from McgR32 tumor-bearing mice with or without MPSSS stimulation were viewed with bright-field illumination, (original magnification ×200) and (B) after Wright’s staining for nuclei (original magnification ×200). (C) Electrical impedance measured using a real-time cell electronic sensing (RT-CES) system. (D) All adherent splenocytes from McgR32 tumor-bearing mice were cultured with or without MPSSS for 72 hours and the expression of MHC I, MHC II, F4/80, CD11c or CD86 on MDSCs were analyzed by FACS. (E) Immunofluorescent staining of F4/80 on MPSSS-treated MDSCs (original magnification ×100). (F) Purified MDSCs were stimulated with MPSSS for 4 hours, and the expression of CXCL9, IL-6, or TGF-β mRNA was determined by RT-PCR. Results were given relative to β-actin mRNA and are presented as means ± SD of triplicate samples from one representative experiment. Data is analyzed with Student's *t-*tests. *P<0.05, **P<0.01.

To contribute the effect of MPSSS against tumor progression to its influence on MDSC differentiation, we investigated its effect on purified MDSCs in vitro. The Ly6C^+^CD11b^+^ cells were sorted from the spleen of McgR32 tumor-bearing mice by FACS, and stimulated with MPSSS or PBS for 3 days. Interestingly, the morphology of MDSCs altered significantly after MPSSS treatment. Untreated cells were mainly round, whereas MPSSS-treated cells exhibited an elongated and irregular form, with long pseudopodia, suggesting that MPSSS induces MDSC differentiation (Fig. 3A). Moreover, the nuclear morphology of MDSCs was also changed as shown in Fig. 3B. In the control group, a large percentage of MDSCs exhibited a “ring-like” nucleus, while in the MPSSS-treated group the ring-like structure disappeared, and instead there were more cells containing multi-nuclei.

A real-time cell electronic sensing system (RT-CES) was used to measure the biophysical characters of MDSCs [13], and the results showed that MPSSS induced a dose-dependent increase in the electrical impedance of MDSCs (Fig. 3C).

Furthermore, the expression of MHC II and F4/80 was up-regulated, CD86 down-regulated though the expression of CD11c and MHC I showed no significant changes after MPSSS treatment (Fig. 3D, E). Interestingly, the expression of CXCL9 was increased, while the expression of IL-6 was reduced after MPSSS stimulation (Fig. 3F). Together, the results indicated that MPSSS promoted the differentiation of MDSCs into more mature macrophages.

### MPSSS Eliminated the MDSC-mediated T cell Inhibition

**Figure 4:**
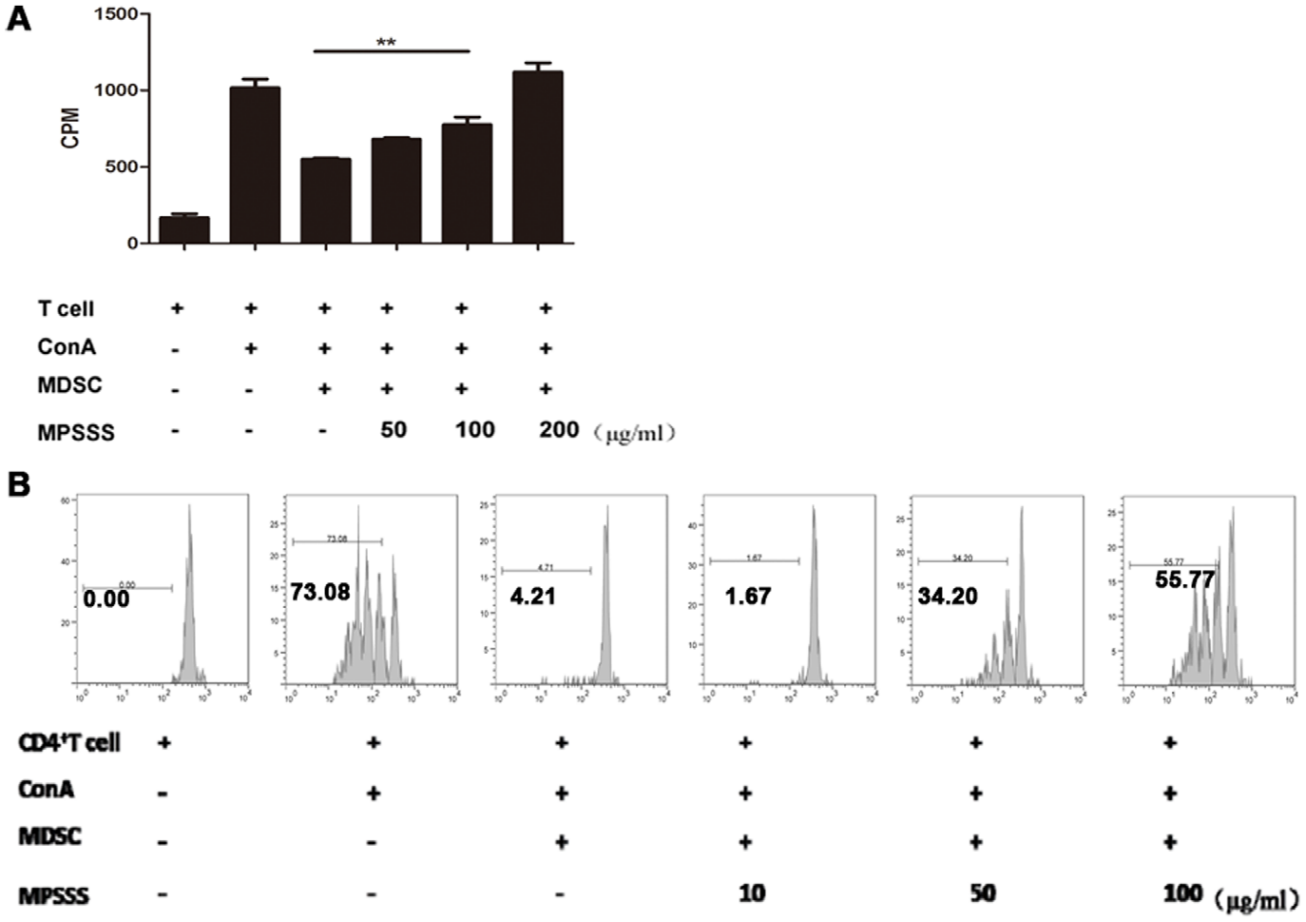
MPSSS promoted functional recovery of splenocytes following MDSC-induced suppression. (A) [^3^H]-thymidine or (B) CFSE-labeled splenocytes were co-cultured with purified MDSCs isolated from McgR32 tumor-bearing mice and stimulated with or without ConA or MPSSS as indicated. Results were presented as mean ± SD of triplicate samples from one representative experiment and were analyzed by one-way ANOVA. Comparisons between these groups were performed using the S-N-K method. **P<0.01.

Although the morphology of MDSCs changed markedly after MPSSS stimulation (Fig. 3A, B), it is still unclear whether their functions were influenced. MDSCs isolated from tumor-bearing mice were co-cultured with a suspension of splenocytes, labeled with [^3^H]-thymidine, and stimulated with ConA in the presence or absence of MPSSS. As shown in Fig. 4A, the T cells proliferation upon ConA stimulation was drastically inhibited by MDSCs; however, when MPSSS was added into the culture system, it recovered in a dose-dependent manner. Similar results were obtained when the 5-(and 6)-carboxyfluorescein diacetate, succinimidyl ester (CFSE) was used to label T cells (Fig. 4B). Therefore, MPSSS was able to eliminate MDSC-mediated T cell inhibition *in vitro*.

### MPSSS Activated Ly6C^high^ CD11b^+^ Cells but not Ly6C^int^ CD11b^+^ Cells

**Figure 5:**
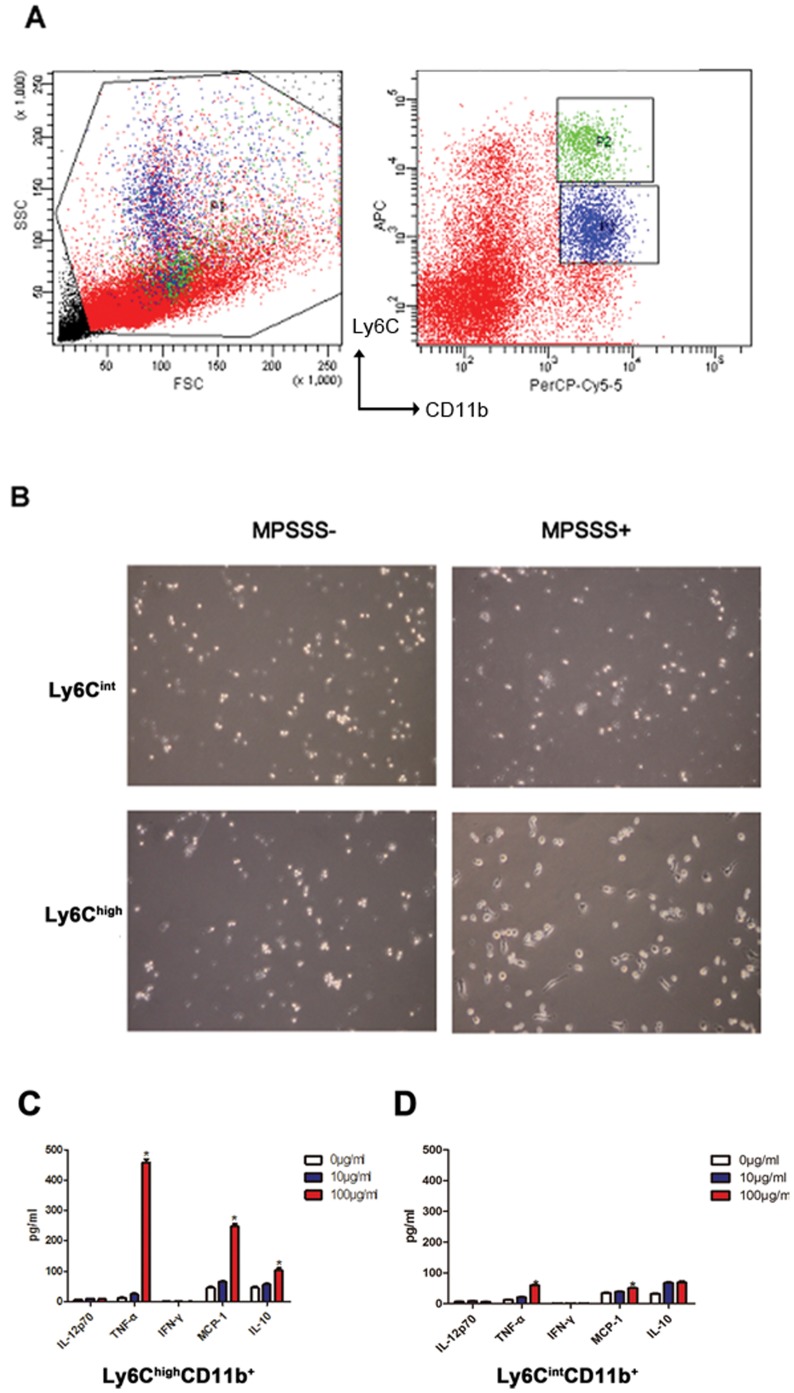
MPSSS activated Ly6C^high^ CD11b^+^, but not Ly6C^int^ CD11b^+^ cells. (A) Ly6C^high^ CD11b^+^ cells (P2) and Ly6C^int^ CD11b^+^ cells (P3) were purified from splenocyte (P1) of McgR32 tumor-bearing mice. (B) Ly6C^high^ CD11b^+^ cells and Ly6C^int^ CD11b^+^ cells with MPSSS stimulation for 72 hours were viewed with bright-field (original magnification ×100). (C & D) Supernatants of Ly6C^high^ CD11b^+^ cells and Ly6C^int^ CD11b^+^ cell cultures were used for determination of the cytokines, including TNF-α, IFN-γ, MCP-1 and IL-10. Quantitative data are presented as means ± SD and are analyzed by one-way ANOVA. Multiple comparisons between groups are performed using the S-N-K method. *P<0.05.

Recently, two major subsets of MDSCs have been identified and categorized by specific cell surface markers: granulocytic MDSCs (G-MDSCs) and monocytic MDSCs (M-MDSCs) (Fig. 5A). In mice, G-MDSCs express CD11b^+^Ly6C^int^, whereas M-MDSCs express CD11b^+^Ly6C^high^. To identify which MDSC subset is targeted by MPSSS, both groups of MDSCs were isolated and stimulated by MPSSS. Results showed that MPSSS stimulated M-MDSCs, but not G-MDSCs to produce a high level of TNF-α or MCP-1(Fig. 5B, C, D). These data indicated that MPSSS activated mainly M-MDSC, but not G-MDSCs.

### MPSSS Activated the MyD88-dependent NF-κB signal Pathway in MDSCs

**Figure 6:**
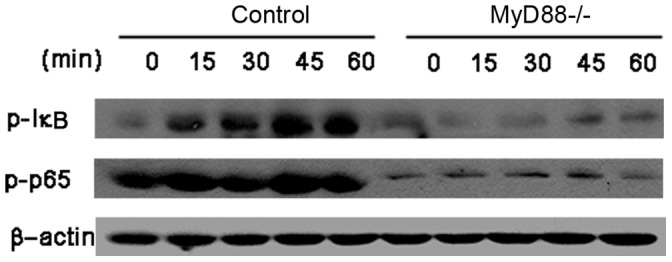
MPSSS activated MDSCs through a MyD88-dependent NF-κB signal pathway. Purified MDSCs from control C57BL/6 and MyD88^−/−^ tumor-bearing mice were stimulated with MPSSS for different times as indicated. Levels of P-p65, and P-IκB in total cell lysates were determined by Western blotting. The expression of β-actin served as a control. The image shown was representative for 3 independent experiments.

To further clarify the mechanism how MPSSS promotes differentiation of MDSCs and maturation of macrophages, we examined the NF-κB signaling pathway in these cells. MPSSS induced the phosphorylation of IκB and p65; However, this was abolished in MDSCs isolated from MyD88^−/−^ mice, suggesting that MPSSS activated MDSCs through a MyD88-dependent pathway (Fig. 6).

## Discussion

Our results demonstrated that MPSSS, a polysaccharide derived from *L. edodes*, did not only stimulate the proliferation of immune cells (Fig. S2), but also induce differentiation of immune suppressor cells. Some polysaccharides are used as natural anti-tumor health supplements which stimulate the proliferation of immune cells to kill tumor cells [14], [15], which are directly attributable to its structure β-(1/3)-D-glucan. Other polysaccharides down-regulate Treg and elicit potent anti-tumor immune responses [16]–[18]. Here, we find that MPSSS increases the ratio of F4/80 on the surface of MDSCs, and an increase in MHC II suggests that antigen presentation ability is enhanced. “Ring” cells (Fig. 3B) have been identified as BM-derived MDSCs [19] and characterized as Gr1^low^, CD11b^+^, CD11c^neg^ and F4/80^+^ myeloid cells. Following MPSSS stimulation, MDSCs essentially disappear, whereas a subset of multinuclear cells appears. These results indicate that MPSSS stimulation induces transformation of MDSCs into macrophages, which enhance the anti-tumor immune response. In order to identify MDSC differentiate into M1 or M2 macrophage, we detect the CXCL9 and IL6 mRNA level of MDSC; however, only CXCL9 mRNA level consists with the M1 macrophage markers [20]. Lipopolysaccharide induces macrophage polarization to M1 by activating NF-κB signal pathway [21] through Toll-like receptor and MyD88, so we detect IκB and P65 to study the mechanism of MDSC differentiation after MPSSS stimulation, and the results show that this pathway is activated, and it depends on MyD88. Although the results indicate to differentiate into M1–like macrophage, the exact relationship between this signal pathway and MDSC differentiation after MPSSS stimulation is still required further investigation. In addition, we found the relative mRNA level of TLR2 and TLR9 increased after MPSSS stimulation (Fig. S3), which indicated that TLR2 and TLR9 may correlate with the MPSSS effect on MDSCs.

MDSCs exhibit a strong ability to inhibit T-cell-mediated rejection of tumor transplants [22]. In addition, they have been implicated in non-immunological functions such as the promotion of angiogenesis, tumor invasion and metastasis. We have previously observed suppression of tumor growth when MDSC function was inhibited [23]. Elimination of MDSCs dramatically improved immune responses in tumor-bearing mice and cancer patients. Treatment of normal mice with anti-Gr1 led not only to a significant decrease in tumor growth but also a complete rejection of the tumors in the majority of mice [24]. All-trans-retinoic acid (ATRA), which drives differentiation of MDSCs, significantly prolonged the anti-tumor effect in two mouse tumor models [25], and also improves tetanus toxoid specific T cell responses in patients with metastatic renal cell carcinoma when combined with two cancer vaccines. However, strong side effects limit its clinical application. Similarly, although anti-Gr1 antibody eliminates granulocytes, it increases the risk of infection. Therefore, novel strategies targeting MDSCs are needed. We previously found that endogenous IL-4 inhibits tumor growth of MCA205 fibrosarcoma by promoting MDSC differentiation into DCs [26], [27], while IL-4 has no effect on late stage tumor growth owing to down-regulation of surface IL-4 receptors on MDSCs [23]. It is therefore necessary to explore novel biological molecules that induce MDSC differentiation.

Polysaccharides isolated from botanical sources such as fungi, algae, lichens and higher plants have also attracted a great deal of attention in biomedicine because of their broad spectrum of therapeutic properties and relatively low toxicity. The mechanisms by which botanicals mediate their effects include modulation of innate immunity, especially macrophage function. However, to the best of our knowledge, there are no data indicating that polysaccharides induce MDSC differentiation.

The observed reduction in immune suppressor cells and the corresponding increase in immune enhancement may be a novel mechanism explaining how polysaccharides from *L. edodes* inhibit tumor growth (Fig. 2A). Moreover, our results indicate that T cell inhibition by MDSCs is reversed, at least in part, in a dose-dependent manner. Our results suggest that polysaccharides may modulate the negative immune regulation, and enhance anti-tumor immunity, which is important for clinical therapy.

### Conclusion

MPSSS, a polysaccharide from *L. edodes*, induced the differentiation of MDSCs, reversed their immune suppressor functions and therefore inhibited tumor growth. This finding may have implications for anti-cancer therapy.

## Materials and Methods

### Purification of Compounds from *L. edodes* Polysaccharides

The fruit body of *L. edodes* was cultured and collected from Suizhou, Hubei province, China. It was identified as *L. edodes* Pegler (Berk.) by Hongwei Liu (COA No: 2010–131) from the Institute of Microbiology, Chinese Academy of Sciences. The fruit body of *L. edodes* was extracted twice with boiling water for two hours at a ratio of 20∶1 (w/w)followed by centrifuging at 5000 rpm×5 min. Supernatants were combined and concentrated to 1/5 of the original volume, and precipitated by adding 95% ethanol (4 volumes). After centrifugation, the precipitate was dried under vacuum conditions to obtain crude polysaccharides. The procedure for extraction and fractionation of the crude polysaccharide is shown in Fig. S1. The structure of MPSSS was determined by use of ^13^C NMR. The ^13^C NMR spectra were obtained on a spectrometer at 500 MHz (Agilent DD2, CA, USA). Samples (20 mg) were dissolved in D_2_O (1 mL, 99.8%) with overnight stirring at room temperature. Spectra were recorded at 25°C after 9,280 scans.

The molecular weight of MPSSS was determined by HPLC. High performance gel permeation chromatography was carried out at 45°C using a Shodex SB-804 column coupled to Shodex SB-802.5 column connected to a Waters 515 HPLC system. 5 microliters of sample (5 mg/mL) was injected, eluted with 0.1 M NaCl and 0.02 M NaN_3_ at a flow rate of 0.5 mL/min and monitored using a refractive index Wyatt-Optilabrex detector coupled to a Wyatt-DAWN HELEOS-II Laser Light Scattering Spectrometer.

### Mouse Tumor Models

C57BL/6 and BALB/c mice were purchased from the Weitong Lihua Company (Beijing, China). *myd*88^−/−^ mice were originally from S. Akira (Osaka University, Osaka, Japan)and were backcrossed to C57BL/6 mice for 10 generations. All mice were maintained in a specific pathogen-free environment at the Institute of Biophysics, Chinese Academy of Sciences. Sex- and age-matched mice were used with the approval of the appropriate authorities. To generate tumor models, 1×10^6^ McgR32 tumor cells (McgR32 cells are fibrosarcoma derived from the MCA205 tumor cell line, and are deficient in IFN-γ receptors, which are helpful for tumor growth. The cell line was constructed in our laboratory) were injected subcutaneously in the flank of C57BL/6 and MyD88^−/−^ mice, and splenocytes were isolated after 20 days. Tumor growth was monitored every 2–5 days and tumor volume was calculated by (length×width×height). To generate tumor metastasis models, 3×10^5^ B16F10 tumor cells were injected into the tail vein, followed by intra-peritoneal injection of MPSSS (25 mg/kg/day) or PBS once a day. On day 15, peripheral blood was used for analysis. To generate an adoptive transfer model, 1×10^7^ splenocytes isolated from GFP^+^ McgR32 tumor-bearing mice were injected into naive C57BL/6 mice through the tail vein. After 4 hours, MPSSS (25 mg/kg) or PBS was injected into the tail vein. Peripheral blood of naive mice was used for FACS analysis after 24 hours of MPSSS/PBS injection.

### Flow Cytometry

Cells were labeled for immunofluorescence and analyzed by flow cytometry for cell surface and/or intracellular molecules. Antibodies including PerCP-anti-CD11b, PerCP-anti-F4/80, APC-anti-CD11c, PE-anti-Ly6C, PE-anti-CD86, PE-anti-MHC II, PE-anti-MHC I (Biolegend, Cambridge, UK) and APC-CD11b (Sungene Biotech, Tianjin, China) were diluted in PBS with 2% FCS (HyClone, Logan, UT, USA).

### In vitro Proliferation Assay

[^3^H] thymidine incorporation was measured as described previously [25]. Briefly, T cells were seeded in 96-well plates at equal density (3×10^5^ cells/ml) and co-cultured with adhesion splenocytes isolated from McgR32 tumor-bearing mice at a ratio of 5∶1. Different concentrations of MPSSS and 0.5 µg/ml ConA were then added. After incubation for 56 hours in a 5% CO_2_ humidified environment at 37°C, [^3^H]-thymidine (1 µCi/well) was added and cells were incubated for an additional 16 hours. After washing twice with cold PBS, cells were counted in a scintillation counter. Each concentration was replicated in triplicate, and at least three independent experiments were performed.

For CCK-8 assays, splenocytes were seeded in 96-well plates at equal density (3×10^5^ cells/ml) in RPMI medium. Different concentration of MPSSS, 10 µg/ml, 50 µg/ml and 100 µg/ml, were added in the medium. After 20 hours, 10 µl of CCK-8 solution (DojinDo Laboratories, Kumamoto, Japan) was added to fresh medium, and cells were incubated further for 4 hours. Absorbance at 450 nm was determined using a microplate reader (BIO-RAD Laboratories, Philadelphia, PA, USA). Results are expressed as a percentage of the absorbance of control cultures. All experiments were performed in triplicate.

For CFSE labeling and proliferation assays, splenocytes were isolated from naive mice and incubated in RPMI 1640 for 4 hours. Suspension cells were then collected and stained with 0.5–1 µM carboxyfluorescein diacetate and CFSE. CFSE-labeled splenocytes were co-cultured at a 5∶1 ratio with MDSCs, stimulated with 0.5 µg/ml ConA, and sorted by FACS Aria III (BD Biosciences, San Diego, CA, USA). After 3 days, cells were stained with CD4 (Biolegend, Cambridge, UK), and the CFSE signal of gated lymphocytes was analyzed.

### Cell and Nuclear Morphology

MDSCs were sorted from McgR32 tumor-bearing mice by FACS with markers for Ly6C and CD11b and stimulated with 10 ng/ml IL-4 and 10 ng/ml GM-CSF with or without MPSSS (100 µg/ml) for 3 days. Adherent cells were digested in 5 mM EDTA, re-suspended to 2×10^6^ cells/ml in 50 µl PBS and centrifuged in a cytospin (Thermo Scientific, Waltham, MA, USA). Cells were then stained with Wright’s stain for 15 minutes and washed with water prior to analysis.

### Electrical Impedance of MDSCs

The RT-CES system (ACEA Biosciences Inc., San Diego, CA, USA) is comprised of three components: an electronic sensor analyzer, a device station and a 96-well strip. The cell culture conditions on the 96× sensor device were the same as those used for tissue culture. Growth media (100 µl) was gently dispensed into a 96-well strip for background readings by the RT-CES system prior to adding to 200 µl of cells at 1×10^6^ cells/ml.

Devices containing cells were kept at room temperature in a tissue culture hood for 15 minutes prior to insertion into the RT-CES device. Cell-sensor impedance was displayed as an arbitrary unit called the cell index.

The cell index at each time point is defined according to the formula:
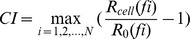
where N is the number of frequency points at which the impedance is measured (i.e., N = 3 for 10 kHz, 25 kHz and 50 kHz), and R_cell_(f_i_) and R_0_(f_i_) are the frequency-dependent electrode resistances with or without cells present in the wells, respectively [13].

Before the start of the experiment, the media in each well was changed to RPMI 1640 and equilibrated for 2 hours. Cells were then incubated in the presence or absence of different concentrations of MPSSS.

### Immune Fluorescence Assay

MDSCs (Ly6C^+^ CD11b^+^ cells) were sorted from spleens of McgR32 tumor-bearing mice and maintained on glass slides with or without 100 µg/ml MPSSS stimulation for 3 days. The mAb used for staining was anti-F4/80 monoclonal antibody (BD Biosciences). Alex Fluor 555-conjugated goat anti-rat IgG (H+L) (Invitrogen, Carlsbad, CA, USA) was used as a secondary antibody. Cell nuclei were counterstained with 4′, 6-diamidino-2-phenylindole. Photos of cells were taken using a digital microscope (OLYMPUS, Tokyo, Japan).

### Real Time RT-PCR

Total RNA was extracted from 5×10^6^ isolated CD11b^+^Gr1^+^ cells stimulated by MPSSS with TRIzol (Invitrogen, Carlsbad, CA, USA) and quantified on a ND-1000 spectrophotometer (NanoDrop Technologies, Wilmington, DE, USA). cDNA was synthesized using 2 µg RNA, 9 nt random primer (TaKaRa, Ohtu, Japan) and M-MLV reverse transcriptase (Promega, Heidelberg, Germany). The amounts of TLR2, TLR4, TLR9, CXCL9, IL-6 and TGF-β mRNA were determined using iQTM SYBR Green Supermix on a MyiQTM system (Bio-Rad Laboratories). β-actin mRNA was used as internal control. The specific primers were:

TLR2∶5'-CTCTTCAGCAAACGCTGTTCT-3'; 5'-GGCGTCTCCCTCTATTGTATTG-3';

TLR4∶5'-CCTGATGACATTCCTTCT-3'; 5'-AGCCACCAGATTCTCTAA-3';

TLR9∶5'-ATGGTTCTCCGTCGAAGGACT-3'; 5'-GAGGCTTCAGCTCACAGGG-3';

IL-6∶5'-ATGGATGCTACCAAACTGGAT-3'; 5'-TGAAGGACTCTGGCTTTGTCT-3';

CXCL9∶5'-TGCTACACTGAAGAACGGAGAT-3'; 5'-TCCTTGAACGACGACGACTT-3';

TGF-β: 5'-CAACAATTCCTGGCGTTACCT-3'; 5'-TGTATTCCGTCTCCTTGGTTCA-3';

actin: 5'-GAAGTGTGACGTTGACATCCGTA-3'; 5'-CTCAGGAGGAGCAATGATCTTGA-3'.

Primers for IL-6, CXCL9 and TGF-β were purchased from Sangon Biotech Shanghai Co., Ltd.

### Western Blotting

Cells were lysed by RIPA solution [50 mM Tris-HCl (pH 7.5), 150 mM NaCl, 1.0% Nonidet P-40, 0.5% (w/v) sodium deoxycholate, 0.1% (w/v) SDS, 1 mM EDTA] supplemented with 100 µM phenylmethanesulfonyl fluoride, 25 µg/ml aprotinin, 1 mM sodium orthovanadate and 50 mM NaF. Aliquots of cell extracts were resolved on 10% SDS-PAGE gel and then transferred to a nitrocellulose membrane (GE Healthcare, Milwaukee, WI, USA) using a semi-dry transfer apparatus (Bio-Rad Laboratories). The primary antibodies used were: Phospho (P)-NF-κB p65 (Ser536; 93H1), and P-IκB (S32; 14D4; Cell Signaling Technology), all of which were diluted by 1∶1000, and β-actin (1∶8000, Sigma-Aldrich, St. Louis, Missouri, USA). HRP-conjugated goat anti-mouse or goat anti-rabbit IgG (Thermo) were used as secondary antibodies. After washing with PBST, the membrane was incubated with chemiluminescent substrate (Thermo) for 5 minutes. Specific bands were visualized by exposing the membrane to X-ray film (Kodak, Rochester, NY, USA) in a dark room.

### Cytokine Detection

Ly6C^high^ CD11b^+^ and Ly6C^int^ CD11b^+^ cells were sorted from spleens of McgR32 tumor-bearing mice and cultured with or without 100 µg/ml MPSSS stimulation for 3 days. Supernatant were analyzed by cytometric bead arrays (CBA, BD Biosciences) in accordance with the manufacturer’s instructions. Briefly, five bead populations with distinct fluorescence intensities were coated with capture antibodies specific for IL-10, MCP-1, IFN-γ, TNF-α and IL-12p70 proteins. The five bead populations were mixed together to form the CBA, which was resolved in the FL3 channel. The capture beads, PE-conjugated detection antibodies and recombinant standards or test samples were incubated together to form sandwich complexes. Following data acquisition, flow cytometry results were generated using CBA Analysis Software (BD Biosciences).

### Statistical Analysis

Data are analyzed using Student's *t* tests, or one-way ANOVA, and are presented as means ± SD. Differences are considered to be statistically significant when *P*<0.05.

## Supporting Information

Figure S1 **Purification of polysaccharides from** ***L. edodes*** **.** We obtained three pure compositions: MPPP, MPSSP2 and MPSSS.
(TIF)

Figure S2 **MPSSS promotes splenocyte proliferation.** Splenocytes isolated from naive C57BL/6 mice (A) or McgR32 tumor-bearing mice (B) were seeded on 96-well plates and stimulated with different concentrations of MPSSS. The product of CCK-8 assay formazan was measured with optical density 450 nm. Results presented are mean ± SD of triplicate samples from one representative experiment. *P<0.05, **P<0.01.
(TIF)

Figure S3 **MPSSS increases the mRNA level of TLR2 and TLR9.** MDSCs were isolated by FACS and stimulated with MPSSS for 0, 3, 6 hours and then the expression of TLR2/4/9 were measured by real-time PCR. Results presented are mean ± SD of triplicates from one representative experiment. *P<0.05, ***P<0.001.
(TIF)

Table S1 **The relationship between ^13^C NMR chemical shift and the corresponding C.**
(DOCX)

## References

[1] Almand B, Clark JI, Nikitina E, van Beynen J, English NR, et al (2001) Increased production of immature myeloid cells in cancer patients: a mechanism of immunosuppression in cancer. J Immunol 166: 678–689. 11123353 10.4049/jimmunol.166.1.678

[2] Gabrilovich D (2004) Mechanisms and functional significance of tumour-induced dendritic-cell defects. Nat Rev Immunol 4: 941–952. 15573129 10.1038/nri1498

[3] Gabrilovich DI, Bronte V, Chen SH, Colombo MP, Ochoa A, et al.. (2007) The terminology issue for myeloid-derived suppressor cells. Cancer Res 67: 425; author reply 426. 10.1158/0008-5472.CAN-06-3037 PMC1941787 17210725

[4] Gallina G, Dolcetti L, Serafini P, De Santo C, Marigo I, et al (2006) Tumors induce a subset of inflammatory monocytes with immunosuppressive activity on CD8+ T cells. J Clin Invest 116: 2777–2790. 17016559 10.1172/JCI28828 PMC1578632

[5] Melani C, Chiodoni C, Forni G, Colombo MP (2003) Myeloid cell expansion elicited by the progression of spontaneous mammary carcinomas in c-erbB-2 transgenic BALB/c mice suppresses immune reactivity. Blood 102: 2138–2145. 12750171 10.1182/blood-2003-01-0190

[6] Yang L, DeBusk LM, Fukuda K, Fingleton B, Green-Jarvis B, et al (2004) Expansion of myeloid immune suppressor Gr+CD11b+ cells in tumor-bearing host directly promotes tumor angiogenesis. Cancer Cell 6: 409–421. 15488763 10.1016/j.ccr.2004.08.031

[7] Chihara G, Maeda Y, Hamuro J, Sasaki T, Fukuoka F (1969) Inhibition of mouse sarcoma 180 by polysaccharides from Lentinus edodes (Berk.) sing. Nature 222: 687–688. 5768289 10.1038/222687a0

[8] Chihara G, Hamuro J, Maeda Y, Arai Y, Fukuoka F (1970) Fractionation and purification of the polysaccharides with marked antitumor activity, especially lentinan, from Lentinus edodes (Berk.) Sing. (an edible mushroom). Cancer Res 30: 2776–2781. 5530561

[9] Hamuro J, Rollinghoff M, Wagner H (1980) Induction of cytotoxic peritoneal exudate cells by T-cell immune adjuvants of the beta(1 leads to 3) glucan-type lentinan and its analogues. Immunology 39: 551–559. 6966608 PMC1458028

[10] Ladanyi A, Timar J, Lapis K (1993) Effect of lentinan on macrophage cytotoxicity against metastatic tumor cells. Cancer Immunol Immunother 36: 123–126. 8425209 10.1007/BF01754412 PMC11038637

[11] Maeda YY, Chihara G (1973) The effects of neonatal thymectomy on the antitumour activity of lentinan, carboxymethylpachymaran and zymosan, and their effects on various immune responses. Int J Cancer 11: 153–161. 4597986 10.1002/ijc.2910110118

[12] Peter G, Karoly V, Imre B, Janos F, Kaneko Y (1988) Effects of lentinan on cytotoxic functions of human lymphocytes. Immunopharmacol Immunotoxicol 10: 157–163. 3171104 10.3109/08923978809014330

[13] Solly K, Wang X, Xu X, Strulovici B, Zheng W (2004) Application of real-time cell electronic sensing (RT-CES) technology to cell-based assays. Assay Drug Dev Technol 2: 363–372. 15357917 10.1089/adt.2004.2.363

[14] Zhang Y, Li S, Wang X, Zhang L, Cheung PCK (2011) Advances in lentinan: Isolation, structure, chain conformation and bioactivities. Food hydrocolloids 25: 196–206.

[15] Jung HY, Bae IY, Lee S, Lee HG (2011) Effect of the degree of sulfation on the physicochemical and biological properties of< i>Pleurotus eryngii</i>polysaccharides. Food hydrocolloids 25: 1291–1295.

[16] Qi C, Cai Y, Gunn L, Ding C, Li B, et al (2011) Differential pathways regulating innate and adaptive antitumor immune responses by particulate and soluble yeast-derived beta-glucans. Blood 117: 6825–6836. 21531981 10.1182/blood-2011-02-339812 PMC3128477

[17] Ochoa-Reparaz J, Mielcarz DW, Wang Y, Begum-Haque S, Dasgupta S, et al (2010) A polysaccharide from the human commensal Bacteroides fragilis protects against CNS demyelinating disease. Mucosal Immunol 3: 487–495. 20531465 10.1038/mi.2010.29

[18] Li XB, He XJ, Liu B, Xu L, Ju DH, et al (2010) [Immunoregulatory function of Radix Glycyrrhizae polysaccharide in tumor-bearing mice]. Zhong Xi Yi Jie He Xue Bao 8: 363–367. 20388478 10.3736/jcim20100411

[19] Greifenberg V, Ribechini E, Rossner S, Lutz MB (2009) Myeloid-derived suppressor cell activation by combined LPS and IFN-gamma treatment impairs DC development. Eur J Immunol 39: 2865–2876. 19637228 10.1002/eji.200939486

[20] Martinez FO, Gordon S, Locati M, Mantovani A (2006) Transcriptional profiling of the human monocyte-to-macrophage differentiation and polarization: New molecules and patterns of gene expression. Journal of Immunology 177: 7303–7311. 10.4049/jimmunol.177.10.7303 17082649

[21] Lawrence T, Natoli G (2011) Transcriptional regulation of macrophage polarization: enabling diversity with identity. Nat Rev Immunol 11: 750–761. 22025054 10.1038/nri3088

[22] Wang Z, Jiang J, Li Z, Zhang J, Wang H, et al (2010) A myeloid cell population induced by Freund adjuvant suppresses T-cell-mediated antitumor immunity. J Immunother 33: 167–177. 20145547 10.1097/CJI.0b013e3181bed2ba

[23] Jiang J, Wang Z, Li Z, Zhang J, Wang C, et al (2010) Early exposure of high-dose interleukin-4 to tumor stroma reverses myeloid cell-mediated T-cell suppression. Gene Ther 17: 991–999. 20410929 10.1038/gt.2010.54

[24] Kusmartsev S, Cheng F, Yu B, Nefedova Y, Sotomayor E, et al (2003) All-trans-retinoic acid eliminates immature myeloid cells from tumor-bearing mice and improves the effect of vaccination. Cancer Res 63: 4441–4449. 12907617

[25] Mirza N, Fishman M, Fricke I, Dunn M, Neuger AM, et al (2006) All-trans-retinoic acid improves differentiation of myeloid cells and immune response in cancer patients. Cancer Res 66: 9299. 16982775 10.1158/0008-5472.CAN-06-1690 PMC1586106

[26] Li Z, Jiang J, Wang Z, Zhang J, Xiao M, et al (2008) Endogenous interleukin-4 promotes tumor development by increasing tumor cell resistance to apoptosis. Cancer Res 68: 8687–8694. 18974110 10.1158/0008-5472.CAN-08-0449

[27] Li Z, Chen L, Qin Z (2009) Paradoxical roles of IL-4 in tumor immunity. Cell Mol Immunol 6: 415–422. 20003817 10.1038/cmi.2009.53 PMC4003035

