# Supplementary figures and images for: Polysaccharide from Lentinus edodes Inhibits the Immunosuppressive Function of Myeloid-Derived Suppressor Cells
Source: PLoS One. 2012 Dec 18;7(12):e51751. doi: 10.1371/journal.pone.0051751 (PMC3525656; doi:10.1371/journal.pone.0051751)

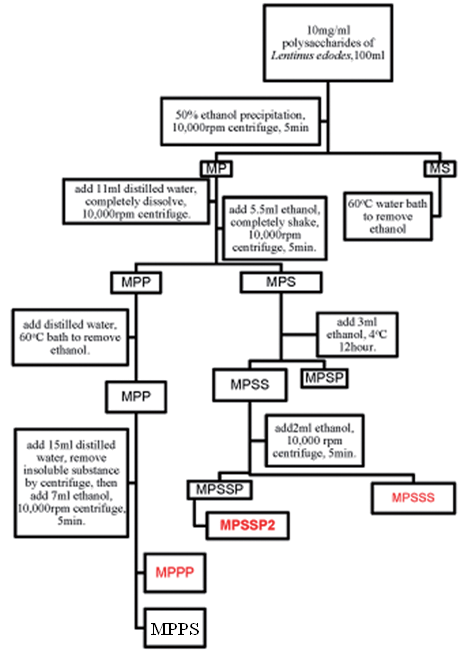

Supplement: Figure S1 — Purification of polysaccharides from L. edodes . We obtained three pure compositions: MPPP, MPSSP2 and MPSSS. (TIF) [file pone.0051751.s001.tif]

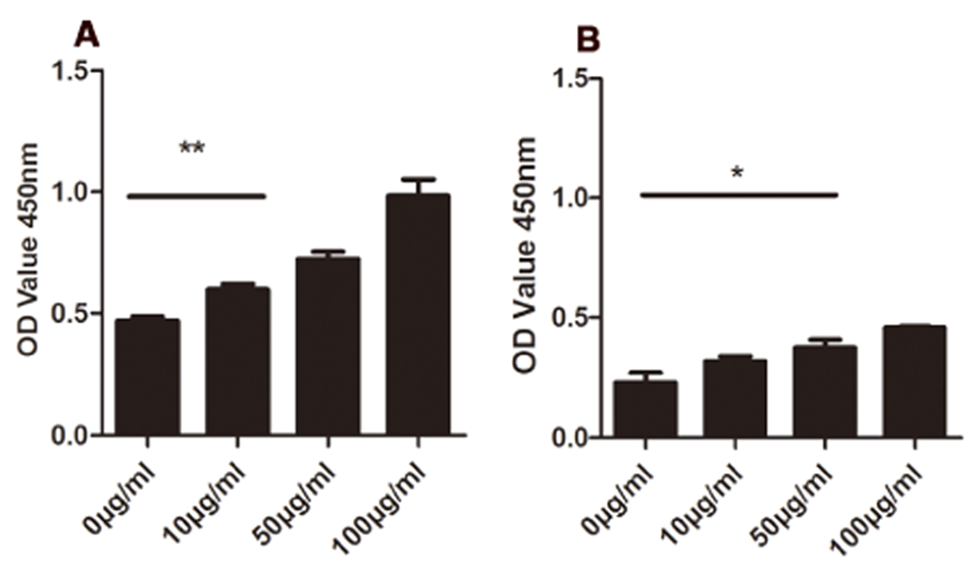

Supplement: Figure S2 — MPSSS promotes splenocyte proliferation. Splenocytes isolated from naive C57BL/6 mice (A) or McgR32 tumor-bearing mice (B) were seeded on 96-well plates and stimulated with different concentrations of MPSSS. The product of CCK-8 assay formazan was measured with optical density 450 nm. Results presented are mean ± SD of triplicate samples from one representative experiment. *P<0.05, **P<0.01. (TIF) [file pone.0051751.s002.tif]

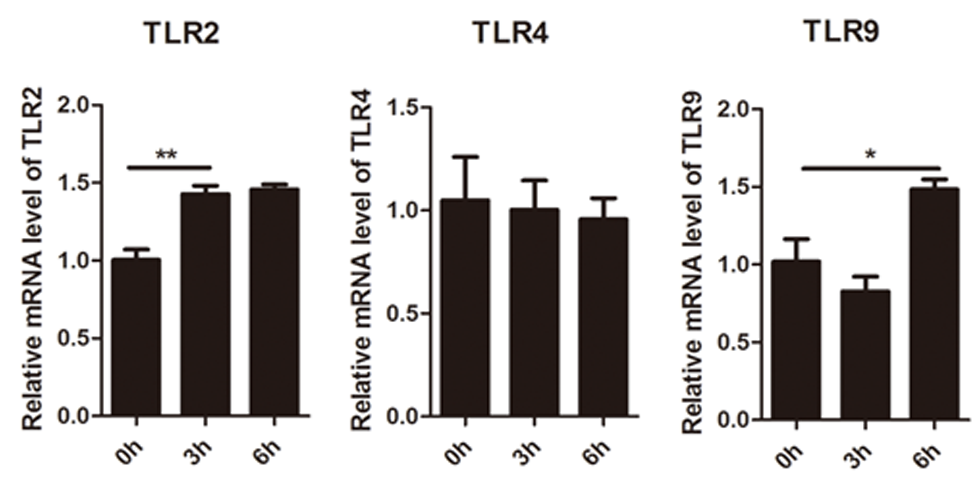

Supplement: Figure S3 — MPSSS increases the mRNA level of TLR2 and TLR9. MDSCs were isolated by FACS and stimulated with MPSSS for 0, 3, 6 hours and then the expression of TLR2/4/9 were measured by real-time PCR. Results presented are mean ± SD of triplicates from one representative experiment. *P<0.05, ***P<0.001. (TIF) [file pone.0051751.s003.tif]
