# Supplementary material for: Polysaccharide from Lentinus edodes Inhibits the Immunosuppressive Function of Myeloid-Derived Suppressor Cells
Source: PLoS One. 2012 Dec 18;7(12):e51751. doi: 10.1371/journal.pone.0051751 (PMC3525656; doi:10.1371/journal.pone.0051751)
Supplement: Table S1 — The relationship between 13C NMR chemical shift and the corresponding C. (DOCX) [file pone.0051751.s004.docx]

|  | C-1 | C-2 | C-3 | C-4 | C-5 | C-6 |
| --- | --- | --- | --- | --- | --- | --- |
| AGlc units | 105.60 | 75.646 | 78.180 | 72.080 | 77.498 | 71.428 |
| BGlc units | 105.40 | 75.449 | 78.590 | 72.976 | 77.179 | 70.882 |
| CGlc units | 105.20 | 76.071 | 78.590 | 72.976 | 77.179 | 63.340 |
